# Editorial: Omics approaches to delineate the role of gut microbiota-derived metabolites in obesity and metabolic disorders

**DOI:** 10.3389/fendo.2024.1436678

**Published:** 2024-06-11

**Authors:** Charalambos Fotakis, Maria Gazouli, Silvia Turroni

**Affiliations:** ^1^Institute of Chemical Biology, National Hellenic Research Foundation, Athens, Greece; ^2^Department of Basic Medical Sciences, Laboratory of Biology, Medical School, National and Kapodistrian University of Athens, Athens, Greece; ^3^Unit of Microbiome Science and Biotechnology, Department of Pharmacy and Biotechnology, University of Bologna, Bologna, Italy

**Keywords:** obesity, gut microbiota, -omics approaches, metabolomics, NAFLD

Obesity is a complex chronic disorder with a multifactorial etiology that involves genetics, hormones, diet, and environmental stimuli (1). It is considered a medical condition that may trigger complications, such as metabolic syndrome, high blood pressure, insulin resistance, low-grade inflammation, heart disease, diabetes, cancer and fatty liver disease. The gut microbiota is implicated in the pathophysiology of obesity and its associated metabolic disorders (2).

In this context, -omics approaches can elucidate molecular signatures related to overweight and obesity phenotypes at the cellular level, and underpin the relationship between the gut microbiota at the family, genus or species level and host metabolism (3).

On these grounds, this Research Topic aims to elucidate the contribution of -omics approaches in framing fluctuations of gut microbiota-derived metabolites in relation to obesity. Additionally, it provides an overview of the pathogenesis of this condition and outlines plausible risk factors that may trigger the onset of metabolic disorders.

The selected articles published in this Research Topic include four research articles and one review.

## Overview of contributions

Two research articles implemented high-throughput, high-resolution analytical techniques, i.e., nuclear magnetic resonance (NMR) spectroscopy and mass spectrometry (MS), to investigate the circulating metabolome of adolescent obesity.

Obesity poses an increased risk for the onset of non-alcoholic fatty liver disease (NAFLD), while the influence of other factors, such as sex, on the incidence and severity of this liver disease has not yet been fully elucidated. Fotakis et al. used an untargeted NMR metabolomics approach on serum samples from normal, overweight and obese patients to investigate the impact of fatty liver disease. This investigation resulted in a sex-specific metabolic bouquet, that could be used for eliciting surrogate markers and generating diagnostic modalities for the distinct stages of NAFLD in each sex. The discriminative metabolic patterns could aid in the development of novel sex-related therapeutic options if a validation scheme ensues in larger and more diverse patient cohorts.

Another metabolomics study by Wu et al. applied untargeted ultra-performance liquid chromatography (UPLC)-MS to explore the causal relationship between adolescent obesity and hypertension. The researchers identified plasma metabolic patterns and pinpointed alterations in relevant metabolic pathways. They highlighted dysregulations in sphingolipid metabolism, purine metabolism, pyrimidine metabolism, phospholipid metabolism, steroid hormone biosynthesis, tryptophan, tyrosine, and phenylalanine biosynthesis. The authors leveraged on the variation of four differential metabolites to propose an obesity-related metabolite score (OMS), which facilitated the prediction of hypertension risk in adolescents. These sensitive biomarkers may enable the early detection of hypertension in adolescents with obesity.

Two other articles (one research and one review) discussed the relationship between gut microbiota composition, obesity and the onset of metabolic disorders, such as diabetes and dysregulation of thyroid function.

To date, human studies have associated gut microbial dysbiosis with obesity and the onset of metabolic disorders, as type 2 diabetes (3). In this vein, the review by Zhang et al. focused on the causal role of gut microbiota in obesity and type 2 diabetes. It provided insights into the evolving landscape of obesity management and the potential of microbiota-based approaches to address this pressing global health challenge. In particular, the review highlighted the literature on the assessment of longitudinal changes in gut microbiota composition and functionality, and provided insight into their relationship with the two most effective anti-obesity treatments, pharmacotherapy and bariatric surgery. Accumulating evidence suggests that gut microbiota composition and function differ between healthy lean subjects and obese patients, and that the microbiota may have an impact on obesity-related diseases, such as insulin resistance, low-grade inflammation, diabetes, and fatty liver disease (4, 5). Many factors affect the gut microbiota homeostasis of gut microbiota, including diet, genetics, circadian rhythms, medications, probiotics, and antibiotics. Then, the review elaborated on plausible steps to prevent/treat obesity by summarizing the function of gut microbiota, focusing on specific bacteria, their metabolites, and strategies to modulate the gut microbiota.

Another research article by Xie et al. attempted to decipher the precise causal relationship between gut microbiota and thyroid function. To this end, the authors facilitated a genome-wide association study of gut microbiota composition in 18,340 participants from 24 cohorts, summary statistics on thyroid hormones and thyroid-stimulating hormone from the Thyroid Omics Consortium and summary statistics on hypothyroidism and hyperthyroidism from the FinnGen R8 release. Using two-sample MR analysis, the authors identified specific gut microbiota taxa that are predicted to have a causal relationship with thyroid function. These, in turn, may serve as useful biomarkers for early disease diagnosis.

Finally, the last research article took a ‘from clinical bedside to bench and back to the clinic’ approach. Specifically, the study by Yue et al. involved mouse models of obesity to mimic the human condition and provide novel biomarkers using an integrated omics approach. The authors investigated whether semaglutide could improve aortic injury in obese C57BL/6J mice, and used a proteomics approach to investigate its molecular mechanism of action. The results of the proteomic analysis highlighted several differentially expressed proteins (DEPs), in three groups, i.e normal diet (NCD), high-fat diet (HFD and high-fat diet + semaglutide (Sema), of which 537 were up-regulated and 322 down-regulated DEPs in the NCD/HFD group, 251 up-regulated DEPs and 237 down-regulated proteins in the HFD/Sema group. Leveraging on Gene Ontology (GO) enrichment analysis of the overlapping DEPs, these differential proteins were mainly found in extracellular matrix signaling pathways, with mainly changes in the genes Coll5a1, Lama4, Sparc. This highlights the impact of semaglutide in improving vascular function and achieving vascular protection.

Tackling the obesity pandemic requires a better understanding of disease mechanisms to develop new evidence-based diagnostics and precision preventive/therapeutic strategies. -Omics approaches have laid the groundwork for a comprehensive understanding of the impact of the gut microbiome on human health by providing insight into the collective metabolic function of the gut microbiota and elucidating what is actually happening in a biological system. With this foundation, a mechanistic model of the system needs to be built to synthetize its complexity to make predictions and, ultimately, to engineer it, to correct unhealthy features and improve long-term health.

## Author contributions

CF: Writing – review & editing, Writing – original draft. MG: Writing – review & editing, Writing – original draft. ST: Writing – review & editing, Writing – original draft.
